# Identification of novel dihydroorotate dehydrogenase (DHODH) inhibitors for cancer: computational drug repurposing strategy

**DOI:** 10.1186/s40360-025-01007-w

**Published:** 2025-10-22

**Authors:** Rahamathtunnisa Rajamohamed, Shanthi Veerappapillai

**Affiliations:** Department of Biotechnology, School of Bio Sciences and Technology, Vellore Institute of Technology, Vellore, Tamil Nadu India

**Keywords:** DHODH, Drug repurposing, Molecular docking, Molecular dynamics

## Abstract

**Background:**

Dihydroorotate dehydrogenase (DHODH) is a crucial enzyme in *de novo* pyrimidine production, initially sought since its disruption is frequently observed in malignancies. DHODH inhibitors have been demonstrated in multiple trials to effectively destroy tumour cells. For instance, leflunomide, teriflunomide and brequinar are currently in practice for DHODH based therapeutics. However, their usage is hampered due to their less efficiency and toxicity issues. Adding together, no studies have reported drug repurposing efforts targeting DHODH.

**Methods:**

To address these challenges, the present study aimed to identify novel and potent DHODH inhibitors through virtual screening, with a distinct focus on repurposing. Initially, 2619 FDA approved molecules were subjected to molecular docking using AutoDock Vina and Molsoft ICM-Pro. Consequently, binding free energy were performed using Uni-GBSA and PRODIGY. Toxicity and cancer cell line activity were assessed using high precision machine learning techniques. In the end, gold standard simulation studies executed to validate the hit compound inhibitory activity against DHODH protein.

**Results:**

The results of our analysis identified two molecules, DB09026 and DB00503, as potent DHODH inhibitors. It is worth noting that the identified compound able to bind with key residues in the DHODH target protein. Moreover, scaffold analysis supports the existence of anti-cancer activity of the identified compounds. In essence, long 100ns molecular dynamic simulation results were also correlates well with the previous results.

**Conclusion:**

Collectively, we hypothesize that both ritonavir and Aliskiren exhibits minimal side effect, it could be of interesting choice for the management of cancer due to its improved potency.

**Clinical trial number:**

Not applicable.

## Introduction

Pyrimidine nucleotide play a crucial role in cell proliferation and synthesis of DNA and RNA precursors [1]. The production of pyrimidine nucleotides involve two significant pathways namely salvage synthesis and *de novo* pyrimidine biosynthesis [2]. *De novo* pyrimidine biosynthesis produces more number of pyrimidines for the cell growth and it is essential in production of RNA and DNA synthesis [3]. One of the key players in *de novo* pyrimidine biosynthesis is dihydroorotate dehydrogenase (DHODH), a redox enzyme that catalyzes the conversion of dihydoorotate to orotate (rate limiting step) [4, 5]. Recently, literature evidence has shown that DHODH is involved in numerous processes, such as cellular metabolism, growth signalling, ferroptosis, transcription, carcinogenesis, and tumour metastasis, in addition to its role in pyrimidine nucleotide production [2]. Notably, elevated DHODH expression has been implicated in the advancement of numerous diseases, including cancer. Consequently, it represents a crucial therapeutic target [6, 7].

In recent years, numerous small-molecule DHODH inhibitors have emerged as promising therapeutic agents, particularly for cancer treatment. Among them, FDA-approved inhibitors such as leflunomide (LEF) and teriflunomide (TFM), originally used to treat autoimmune diseases like rheumatoid arthritis (RA) and multiple sclerosis (MS), respectively, received a black-box warning in 2010 due to their limited anticancer efficacy and risk of acute liver failure [8]. However, brequinar a potent DHODH inhibitor primarily used for cancer treatment [9], demonstrated higher potency than leflunomide (LEF) and teriflunomide (TFM). Although it showed greater efficacy, its clinical development faced setbacks due to concerns over toxicity and limited efficacy in solid tumors, ultimately leading to the failure of clinical trials [10]. Additionally, several small-molecule DHODH inhibitors, including BAY2402234, ASLAN003, PTC299, JNJ74856665, AG-636, and RP7214, have entered clinical trials for cancer treatment [11].

In one of the study, the integration of in vitro and in vivo studies to induce reactive oxygen species production and inhibit DHODH activity. The results showed that naphthol [2,3-d] [1,2,3]-triazole-4-9-dione compounds exhibited antitumor effects [12]. Similarly, the synthesis and evaluation of indoluidin and its derivatives against various cancer cell lines. The study showed that the compounds had a significant antitumor efficacy in A549 xenograft model [13]. Another investigation assessed the teriflunomide derivatives against colorectal cancer using a biphenyl scaffold. The findings revealed that A37 would be an efficient DHODH inhibitor for the management of colorectal cancer [14]. Furthermore, a study reported the link between DHODH enzyme and lysine degradation using a bioinformatics approach and highlighted its importance as a therapeutic target for cancer therapy. Despite the number of recent studies, none of these small molecules have succeeded in clinical trials. These challenges underscore the urgent need for the development of more potent and selective DHODH inhibitors with improved efficacy and safety profiles.

Previous attempts to develop DHODH inhibitors were unsuccessful due to toxicity and low efficacy [8]. Adding together, no studies have reported drug repurposing efforts targeting DHODH. To address these challenges, the present study aimed to identify novel and potent DHODH inhibitors through virtual screening, with a distinct focus on repurposing. In-silico approaches like virtual screening and molecular docking are very significant in the initial stages of discovering new drugs as they facilitate rapid and cost-effective means for the identification of lead compounds [4]. It helps to screen large repositories with high precision. Such an initial screening helps to retrieve potent compounds with less computational cost and ultimately drive the cancer drug therapeutics pipeline [5]. Indeed, this present strategy of combining machine learning with high-end simulation approach offers a promising avenue for overcoming past limitations and advancing DHODH targeted cancer therapy.

## Methods

### Data set curation and docking validation

The 3D structural information of the DHODH protein (PDB ID: 1D3G) in complex with brequinar at a resolution of 1.60 Å was retrieved from the Protein Data Bank and used as the reference compound. A total of 2619 FDA approved compounds were procured from DrugBank repository and utilized for the virtual screening. Initially, the accuracy and reliability of the docking was assessed by employing superimposition technique. For this purpose, complexes were generated both using AutoDock Vina and Molsoft ICM-Pro and the results are analysed based on the superimposed (RMSD) Root Mean Square Deviation.

### Molecular docking

#### Molecular docking using AutoDock Vina

The binding affinity of the protein-ligand complexes was analyzed through molecular docking using AutoDock Vina v1.1.2 and Molsoft ICM-Pro. Prior to docking, hydrogen atoms were incorporated into the protein using AutoDock 4.2.6. All the water molecules and bounded ligands were systematically eliminated from the PDB structure. Subsequently, protein was assigned with Kollman charges and polar hydrogen bonds [15]. The gasteiger charges were assigned, the torsional root was augmented, and subsequently, brequinar was introduced to the target protein [16]. The grid box was generated with center coordinates (x = 49.872, y = 40.422, z = -4.83) around the active site region. Furtherly the compounds were subjected docking analysis using AutoDock Vina v1.1.2. It utilizes Monte Carlo algorithm combined with BFGS (Broyden-Fletcher-Goldfarb-Shanno) gradient-based optimizer [17] for calculating the docking score.

#### Molecular docking using Molsoft ICM pro

The docking analysis was also executed using Molsoft ICM-Pro software to avoid the false positive prediction. The protein structure (1D3G) was turned into ICM objects by removing water molecules and optimising hydrogen bonds. Missing side chains were treated before the docking. The Vander Waals contact of the receptor is utilised to locate pockets for a grid construction [18]. Biased probability Monte Carlo was used in docking to optimize the small molecules internal coordinates [19].

### Binding free energy calculations

The Molecular mechanics/Generalized-Born (Poisson–Boltzmann) surface area (MM/GB(PB)SA), is one of the most popular method, achieved reasonable correlation with experimental affinities was employed to validate the accuracy of the docking results [20, 21]. In the present study, Uni-GBSA and PRODIGY tools were employed to ascertain the ΔG_bind_ of the docked protein - ligand complexes.

Uni-GBSA is an automatic workflow platform used to calculate MM/GBSA from force field building and structure optimize free energy calculation [22]. The binding free energy is determined using the following equation:

$$\Delta\text{G}=\Delta\text{G}_{\text{protein-ligand}}-\Delta\text{G}_{\text{protein}}-\Delta\text{G}_{\text{ligand}}$$

where the energy estimates of the optimized complex (protein-ligand), optimized free ligand, and optimized free protein [23]. The ΔG is estimated as ΔG = ΔH - TΔS, ΔH is expressed as ΔH = ΔE_MM_ + ΔG_solv_. Δ_EMM_ is the differences between the protein ligand complex as the total minimized energies and ΔG_solv_ is the sum of polar and non-polar concentrations of the protein ligand complex. -TΔS represents the protein-ligand complex’s conformational entropy, where T is the absolute temperature are represented respectively [22].

Similarly, PRODIGY-LIG, an online web server, uses a combination of structural properties like inter-molecular electrostatic energy and number of intermolecular atomic contacts from experimental datasets to predict the ΔG bind of protein-ligand complexes [24]. The ∆G_prediction_ is calculated as follows:

$$\begin{aligned}\Delta{\text{G}}_{\text{n}\text{o}\text{e}\text{l}\text{e}\text{c}\text{t}}\:=&\:0.0354707\:\text{*}\:\text{A}\text{C}\text{N}\text{N}\hspace{0.17em}-\hspace{0.17em}0.1277895\:\text{*}\\&\text{A}\text{C}\text{C}\text{C}\hspace{0.17em}-\hspace{0.17em}0.0072166\:\text{*}\:\text{A}\text{C}\text{C}\text{N}\hspace{0.17em}-\hspace{0.17em}5.1923181\end{aligned}$$

where the intermolecular atomic connections (ACs) (within a cutoff of 10.5 Å) is categorised based on the atoms that interact O = Oxygen, C = Carbon, X = other atoms, and N = nitrogen [25, 26].

### In-silico toxicity prediction and interaction analysis

The Protox-II algorithm (https://tox.charite.de/protox3/) was used to assess the potential acute toxic endpoints of the screened lead compounds. The server predicts LD_50_ and evaluates a variety of toxicological endpoints, including organ toxicity, oral toxicity, and toxicity targets [27]. ProTox-II uses molecular similarity, pharmacophore-based, fragment propensities, and machine learning models to predict multiple toxicity endpoints. This in-silico prediction platform is expected to improve the hit selection and optimisation process while also providing fresh insights into the toxicity mechanism [28]. Finally, the binding of ligand in DHODH was evaluated using the protein-ligand interaction profiler server [20].

### In-silico biological activity prediction

The biological anticancer activity of the reference and screened lead compounds were assessed using the pdCSM-cancer tool (https://biosig.lab.uq.edu.au/pdcsm_cancer/prediction) Notably, pdCSM-cancer employs a graph-based signature approach, which has been demonstrated to efficiently represent chemical and biomolecular datasets, as well as to predict pharmacokinetics, toxicity, and bioactivity. Specifically, pdCSM-cancer forecasts the activity of compounds against cancer cell lines based on their graph-derived structural features. The model was developed and validated using clinical data comprising over 18,000 compounds tested across 74 cancer cell lines and 9 tumor types [29]. The literature evidences highlights that Pearson correlation coefficients (r) ranging from 0.70 to 0.85 and root mean square error (RMSE) values between 0.5 and 0.9 for IC_50_ predictions across various cancer cell lines, based on GDSC and CCLE datasets [30]. These metrics underscore the high fidelity of pdCSM-cancer’s predictions when benchmarked against experimental drug sensitivity data. The GI50% was predicted using the SMILES notation of the compounds as input. It predicts the cell-line growth inhibitory action of substances in logarithmic units (log µg/ml) [31].

### Molecular dynamic simulations

The dynamic and adaptable properties of the complexes were investigated at the molecular level using molecular dynamic simulations. In this investigation, MD simulations for the DHODH complexes were carried out using GROMACS version 2020.2. To create a ligand topology, docked ligands were obtained and used with the CHARMM General Force Field (CGenFF). Furthermore, the protein topology was constructed using the CHARMM36 force field. The systems were housed inside a dodecahedron box and solvated using the Simple Point Charge water model. Water molecules were replaced with counter-ions in the solution to provide complete neutrality [20, 27]. Initially, the energy was minimised over 50,000 cycles using the steepest descent approach to resolve steric disputes. Subsequently, the system was further minimised and equilibrated for 1000 ps under the NVT and NPT stages. Using a modified Berendsen thermostat for temperature coupling, a modified Parrinello-Rahman pressure coupling approach, and the V-rescale method, the system was kept at 300 K and 1 bar. After the systems had stabilised, they were subjected to a 100-ns production run at 300 K and 1 bar of pressure. The resultant trajectories were examined using GROMACS tools for several metrics, including radius of gyration (Rg), solvent accessible surface area (SASA), root mean square fluctuation (RMSF), and root mean square deviation (RMSD). The Grace tool was used to graphically visualise these parameters [32].

## Results and discussion

### Docking validation

**Fig. 1:**
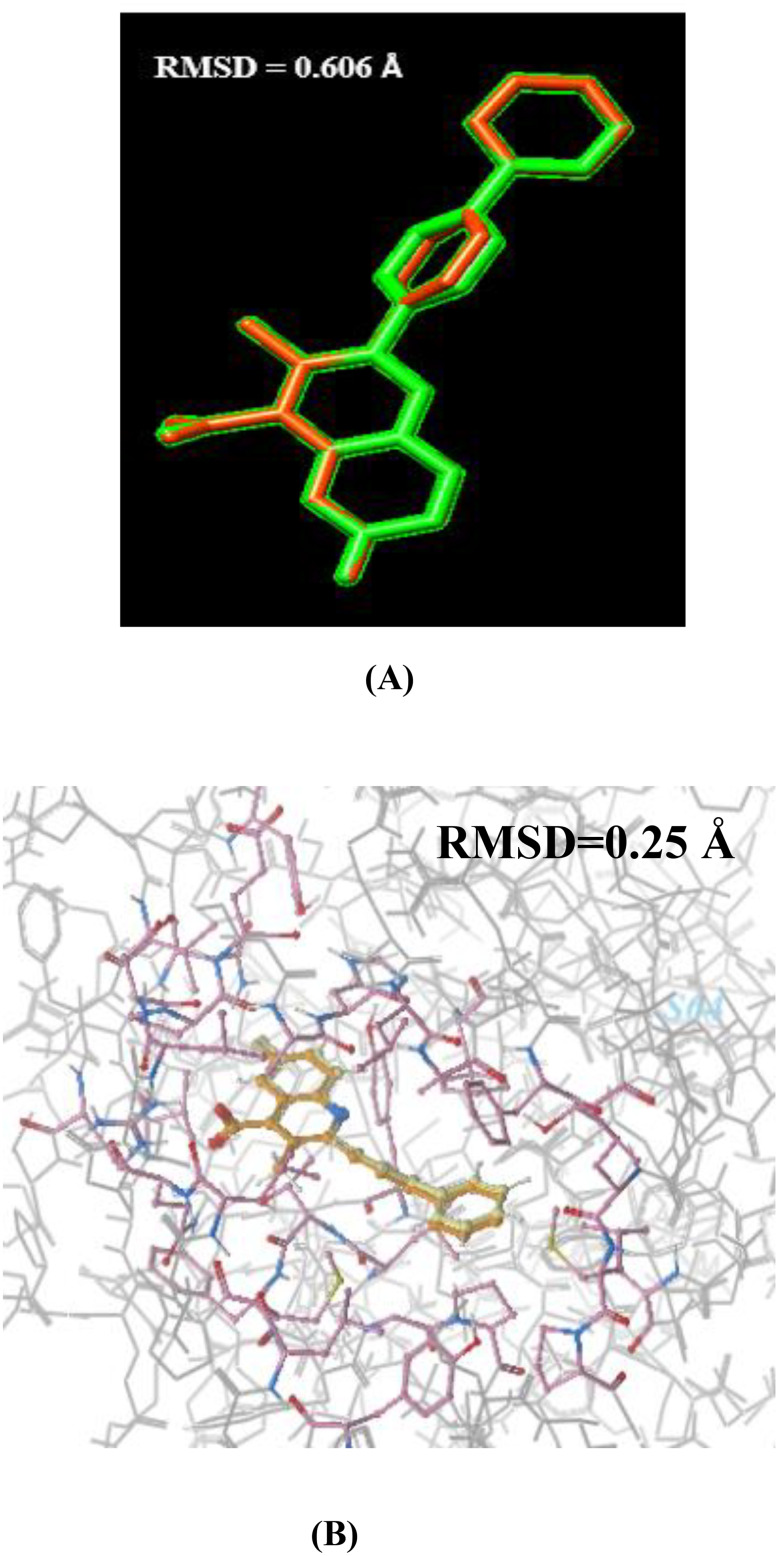
Superimposition of docked brequinar and cocrystallized complex in the active site of 1D3G protein **a**) Superimposition of docked brequinar (red) and cocrystallized complex (green) in the active site of 1D3G protein using AutoDock Vina **b**) Superimposition of docked brequinar (white) and cocrystallized complex (yellow) in the active site of 1D3G protein using Molsoft ICM pro

The docking approach was validated by re-docking the brequinar into the active region of the DHODH protein. The superimposed fit was assessed using the RMSD value between the crystallographic structure and the redocked complexes. The RMSD value of less than 2.0 Å is considered high correlation with experimentally derived structure. Here, the RMSD value was observed to be 0.60 Å and 0.25 Å for AutoDock Vina and Molsoft ICM-Pro respectively. The super imposition of the redocked complex (red) and the co-crystalised complex (green) using AutoDock Vina is shown in Fig. 1a. Redocked complex (white) and co-crystalised complex (yellow) using Molsoft ICM pro is represented in Fig. 1b.

### Molecular docking analysis

The crucial residues in the binding pockets of DHODH were explored from the literature evidences before docking analysis [33]. The active site comprised of hydrophobic residues such as TYR38, MET43, LEU46, LEU50, ALA55, LEU58, ALA59, PHE62, LEU67, LEU68, PRO69, PHE98, MET111, LEU359, and PRO364 alongside ARG136, GLN47, TYR356 and THR360, crucial player in hydrogen bonding interaction [33, 34]. Subsequently, brequinar and 2619 FDA- approved molecules were docked against the binding pocket of DHODH using AutoDock tool and Molsoft ICM-Pro. According to the docking results, the binding affinity of the brequinar was found to be -13.20 kcal/mol in AutoDock Vina and − 16.47 kcal/mol in Molsoft ICM-Pro. The binding affinities of the drug molecules library ranged from − 98.8 to -1.0 kcal/mol (Table 1). The docking results shown that total 920 compounds exhibited better results in the AutoDock Vina and 1027 compounds shown a better results in the Molsoft ICM-Pro. The results of the docking algorithms are integrated to avoid the false positive prediction. This integration yielded a total of 205 compounds that exhibited better binding affinity than brequinar.

**Table 1:** Docking results of the AutoDock Vina and Molsoft ICM pro of reference brequinar and 17 lead molecules

S.no	DrugBank ID	Name	AutoDockVina (kcal/mol)	Molsoft ICM pro (kcal/mol)
1	DB03480	Brequinar	-13.2	-16.47
2	DB00137	Lutein	-17.6	-20.83
3	DB00481	Raloxifene	-16.8	-17.15
4	DB00503	Ritonavir	-16	-31.16
5	DB00549	Zafirlukast	-15	-41.48
6	DB00654	Latanoprost	-17	-58.47
7	DB00944	Demecarium	-18.3	-31.89
8	DB00947	Fulvestrant	-17.3	-34.64
9	DB01100	Pimozide	-15.9	-22.94
10	DB06249	Arzoxifene	-14.3	-17.57
11	DB06401	Bazedoxifene	-16.7	-16.84
12	DB06695	Dabigatran etexilate	-14.3	-42.89
13	DB08909	Glycerol phenylbutyrate	-16.2	-56.50
14	DB08964	Gemeprost	-15.8	-55.31
15	DB09026	Aliskiren	-16.1	-16.68
16	DB09030	Vorapaxar	-16.6	-18.14
17	DB13615	Mifamurtide	-14.2	-48.32
18	DB14185	Aripiprazole lauroxil	-15.8	-45.67

### Binding free energy analysis using Uni-GBSA and PRODIGY

The molecular docking was further rescored by binding free energy analysis using the MM/GB(PB)SA algorithm [35]. The ΔG_bind_ of brequinar was determined to be -67.57 kcal/mol used as a threshold to scrutinise the 205 obtained compounds. It is evident that the 51 compounds exhibits better binding energy profile against DHODH protein.

Further, the binding free energies of 51 compounds were re-evaluated using PRODIGY-LIG tool [26]. The binding free energy of the molecules spanned from − 14.65 to -12.00 kcal/mol. The binding free energy of the brequinar (-10.6 kcal/mol) was used as a threshold to sort the lead molecules. It is important to note that a total 17 molecules showed better binding free energy than the reference. Therefore, these 17 lead compounds were considered for further analysis. The binding free energy calculations are tabulated in Table 2.

**Table 2:** Binding free energy calculations of reference brequinar and 17 lead molecules

S.no	DrugBank ID	Name	(MMGBSA) Molecular mechanics with generalized born and surface area solvation					Prodigy webserver
			Van der Waals (Kcal/mol)	Electrostatic (Kcal/mol)	Solvation (Kcal/mol)	ΔG_bind_ (Kcal/mol)		ΔG prediction (Kcal/mol)
1	DB03480	Brequinar	-63.78	-9.65	5.86	-67.57		-10.6
2	DB00137	Lutein	-81.44	-1.90	-2.77	-86.11		-15.21
3	DB00481	Raloxifene	-70.07	-3.01	0.96	-72.11		-13.25
4	DB00503	Ritonavir	-91.30	-10.03	5.66	-95.67		-14.44
5	DB00549	Zafirlukast	-78.64	-0.76	-0.75	-80.16		-12.12
6	DB00654	Latanoprost	-69.61	-6.37	3.01	-72.98		-13.03
7	DB00944	Demecarium	-87.52	-2.30	-4.58	-94.41		-12.53
8	DB00947	Fulvestrant	-78.16	3.85	-4.94	-79.24		-15.91
9	DB01100	Pimozide	-66.01	0.36	-2.74	-68.40		-12.25
10	DB06249	Arzoxifene	-69.64	-2.52	0.28	-71.89		-13.29
11	DB06401	Bazedoxifene	-71.53	-1.44	0.90	-72.06		-13.28
12	DB06695	Dabigatran etexilate	-100.12	-5.12	5.00	-100.24		-12.00
13	DB08909	Glycerol phenylbutyrate	-84.14	-4.65	2.07	-86.72		-14.65
14	DB08964	Gemeprost	-63.84	-7.97	3.75	-68.05		-12.06
15	DB09026	Aliskiren	-67.19	-11.61	6.77	-72.03		-12.50
16	DB09030	Vorapaxar	-69.86	-3.57	2.12	-71.32		-12.78
17	DB13615	Mifamurtide	-101.30	-18.98	20.64	-99.64		-12.25
18	DB14185	Aripiprazole lauroxil	-91.63	-2.20	-1.73		-87.68	-13.93

### In- silico drug toxicity prediction

The fundamental challenge in lead optimization is distinguishing drug-like molecules from non-drug compounds [27]. The significant toxicological endpoints, such as carcinogenicity, cytotoxicity and mutagenicity, were investigated for both the reference and lead molecules [28]. Table 3 shows the toxicity profile of the brequinar and the lead molecules. The brequinar was seen to be inactive with LD_50_ of 495 mg/kg. The results of 17 lead compounds fall under the toxicity class of II – V with LD_50_ range from 6 mg/kg to 4000 mg/kg. The analysis revealed that 15 lead molecules except DB00944 and DB06249 showed active results in the carcinogenicity and cytotoxicity.

**Table 3:** Toxicity prediction using PROTOX-II webserver for reference brequinar and17 lead molecules

S.no	DrugBank ID	Name	LD50	Toxicity class	Carcinogenicity	Mutagenicity	Cytotoxicity
1	DB03480	Brequinar	495 mg/kg	IV	Inactive	Inactive	Inactive
2	DB00137	Lutein	10 mg/kg	II	Inactive	Inactive	Inactive
3	DB00481	Raloxifene	400 mg/kg	IV	Inactive	Inactive	Inactive
4	DB00503	Ritonavir	1000 mg/kg	IV	Inactive	Inactive	Inactive
5	DB00549	Zafirlukast	300 mg/kg	III	Inactive	Inactive	Inactive
6	DB00654	Latanoprost	50 mg/kg	II	Inactive	Inactive	Inactive
7	DB00944	Demecarium	6 mg/kg	II	active	Inactive	Inactive
8	DB00947	Fulvestrant	2000 mg/kg	IV	Inactive	Inactive	Inactive
9	DB01100	Pimozide	464 mg/kg	IV	Inactive	Inactive	Inactive
10	DB06249	Arzoxifene	2320 mg/kg	V	Inactive	Inactive	active
11	DB06401	Bazedoxifene	200 mg/kg	III	Inactive	Inactive	Inactive
12	DB06695	Dabigatran etexilate	200 mg/kg	III	Inactive	Inactive	Inactive
13	DB08909	Glycerol phenylbutyrate	731 mg/kg	IV	Inactive	Inactive	Inactive
14	DB08964	Gemeprost	57 mg/kg	III	Inactive	Inactive	Inactive
15	DB09026	Aliskiren	4000 mg/kg	V	Inactive	Inactive	Inactive
16	DB09030	Vorapaxar	9 mg/kg	II	Inactive	Inactive	Inactive
17	DB13615	Mifamurtide	1832 mg/kg	IV	Inactive	Inactive	Inactive
18	DB14185	Aripiprazole lauroxil	800 mg/kg	IV	Inactive	Inactive	Inactive

### In-silico anticancer activity analysis

The pdCSM-cancer, a machine learning based online tool was used to predict the biological activity for 17 lead compounds against the most prevalent cancer types including lung, breast, colorectal, and prostate [36]. The pdCSM-cancer algorithm is trained and validated using data from human cancer cell lines (NCI-60). Here, the cancer cell lines such as A549 (lung), MCF7 (breast), HCT116 (colorectal) and PC3 (prostate) considered in the present analysis. The results were analysed in terms of the GI50% (growth inhibitory concentration by 50%) of the compounds. To benchmark our predictions, brequinar was used as a reference compound. The GI₅₀ values of brequinar were found to be 4.43, 4.41, 5.71, and 5.66 in cell lines respectively A549, MCF7, HCT116 and PC3. Notably, brequinar exhibited least activity against HCT116 as observed from its higher GI₅₀ value than studied cell lines. Interestingly, all the screened compounds demonstrated lower GI₅₀ values in HCT116 compared to brequinar, suggesting enhanced anticancer activity against colorectal cells. Zafirlukast and Glycerol Phenylbutyrate showed potency in lung, colorectal and prostate cancer cell lines. None of the screened compounds showed potency against breast cancer cell line, MCF7 in our analysis (Table 4). Thus, we have taken all the compounds for the subsequent interaction analysis.

**Table 4:** Anticancer prediction from PdCSM webserver for reference brequinar and 17 lead molecules

S No	Drug Bank ID	Name	Lung (A549)	Breast (MCF7)	Colorectum (HCT116)	Prostate (PC3)
1	DB03480	Brequinar	4.43	4.41	5.71	5.66
2	DB00137	Lutein	4.44	4.55	**4.45**	**4.63**
3	DB00481	Raloxifene	4.83	5.03	**5.15**	**5.22**
4	DB00503	Ritonavir	4.53	5.44	**4.49**	**5.58**
5	DB00549	Zafirlukast	**4.42**	5.26	**4.61**	**5.17**
6	DB00654	Latanoprost	4.82	4.97	**4.59**	**4.90**
7	DB00944	Demecarium	4.63	5.26	**5.15**	**5.38**
8	DB00947	Fulvestrant	4.74	5.33	**5.35**	**5.07**
9	DB01100	Pimozide	4.81	5.27	**5.07**	**5.07**
10	DB06249	Arzoxifene	5.08	5.02	**5.36**	**5.29**
11	DB06401	Bazedoxifene	4.95	5.12	**5.26**	**5.28**
12	DB06695	Dabigatran etexilate	4.65	5.60	**5.22**	**5.36**
13	DB08909	Glycerol phenylbutyrate	**4.35**	5.03	**4.53**	**4.91**
14	DB08964	Gemeprost	4.52	5.25	**4.79**	**5.19**
15	DB09026	Aliskiren	5.04	5.55	**5.24**	**5.38**
16	DB09030	Vorapaxar	4.76	5.34	**5.04**	**5.32**
17	DB13615	Mifamurtide	**4.16**	5.40	**5.02**	6.41
18	DB14185	Aripiprazole lauroxil	4.81	5.42	**5.22**	**5.17**

The bold highlights the better anticancer activity of the compounds. The predicted GI₅₀ values are expressed in − log₁₀[M]

### Interaction analysis of the protein-ligand complex

**Fig. 2:**
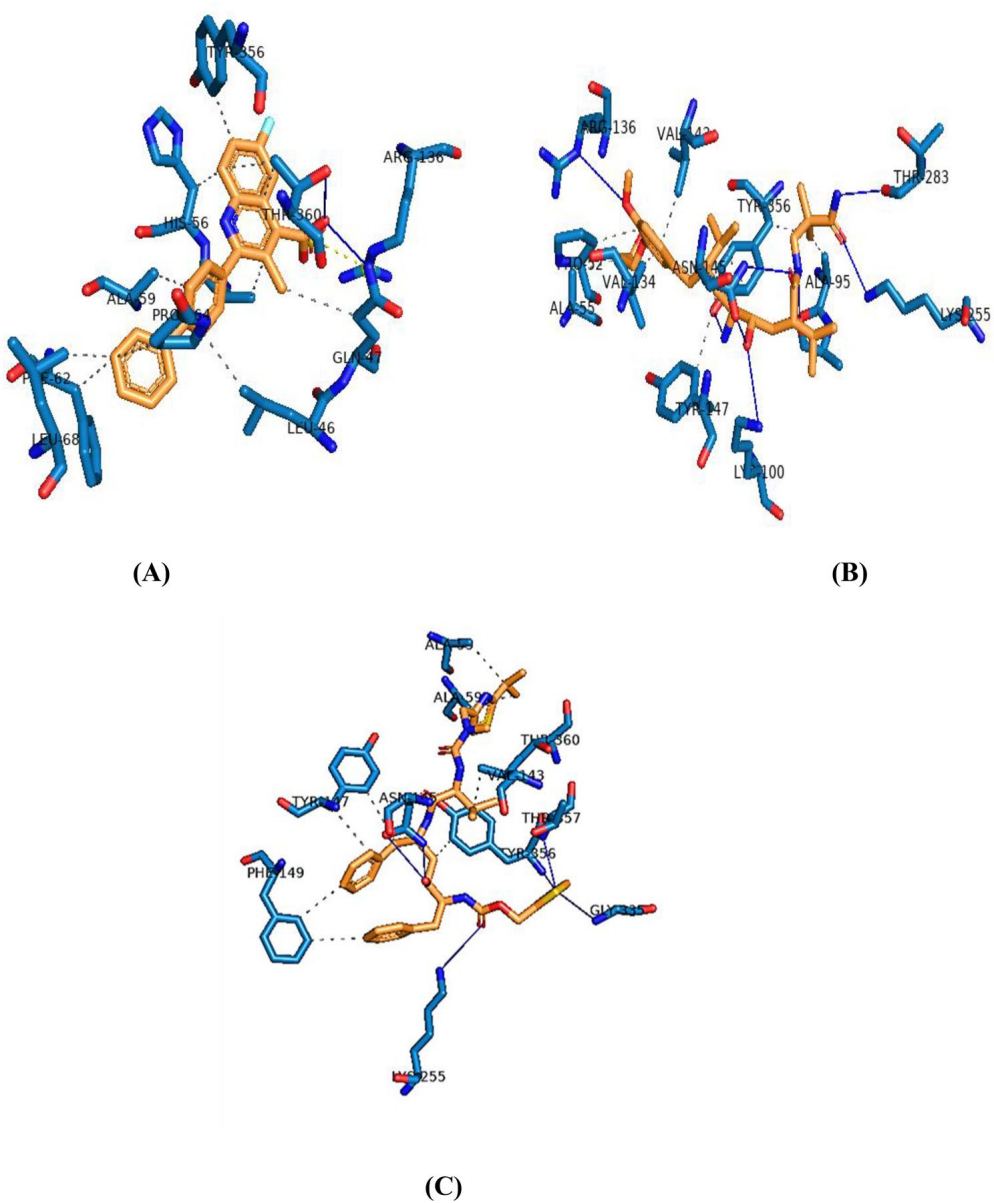
Interaction analysis of **a**) Reference - Brequinar; **b**) DB09026 Aliskiren and c)DB00503 Ritonavir towards the target protein, blue-coloured lines indicate hydrogen bond interactions and grey colour line signifies hydrophobic interactions

The post-molecular docking study was examined to comprehend the binding mechanism of the reference and 17 lead compounds in the active site of DHODH. Figure 2 represents the protein-ligand interaction pattern where hydrogen bond interactions are indicated by the blue, while hydrophobic interactions are indicated by the grey dashed lines [27]. The interaction profiling of DHODH-brequinar showed that it forms hydrogen bonds with GLN47 and THR360 residues, respectively. Besides, LEU46, GLN47, ALA55, HIS56, ALA59, PHE62, LEU68, TYR356, THR360 and PRO364 were able to form hydrophobic interactions. ARG136 forms a salt bridge with the carboxylate group of DHODH. It is likely that TYR356, provides the protons required to from a neutral hydroquinone following electron transfer from FMN [33, 34]. Although there are many binding interactions, hydrogen bonding is one of the key non-covalent interactions in stabilizing the protein-ligand complex [37]. Indeed, these patterns of interactions were explored for all the investigated lead compounds. Among which DB09026, DB00503 and DB13615 showed a high number of hydrogen bonds compared to other compounds. Hydrogen bonds have significant interactions because they can be crucial for drug partition and permeability, structural stability, molecular recognition, and enzyme catalysis. A drug’s solubility makes significant interactions with its biomolecular targets, which can be enhanced by the presence of functional groups that can form hydrogen bonds, promoting strong binding [38]. Further, DHODH-DB09026 was able to form nine hydrogen bonds with ARG136, ALA96, THR283, LYS100, LYS255, TYR356 and ASN145. The presence of TYR356 and ARG136 indicates a strong binding, conforming to the orientation of active site residues closer to the hit molecule. The binding pattern of DHODH-DB00503 involves interactions with ASN145, TYR356, LYS255, GLY335 and THR357, forming seven hydrogen bonds. Ten hydrophobic bonds form with ALA55, ALA59, VAL143, THR360, TYR356, TYR147, and PHE149. Primarily, TYR356 forms a higher number of hydrogen bonds, showing strong hydrogen bonding and facilitates the binding of small molecule with minimal strain of the hits.

Although DB13615 forms two hydrogen bonds with ARG136, GLN47, and ASP51, due to its higher molecular weight DB13615 is not considered a hit compound. Higher molecular weight FDA-approved compounds can lead to issues such as reduced solubility, lower permeability, inconsistent pharmacokinetics and low oral bioavailability [39]. Overall, DB09026 and DB00503 showed a greater number of hydrogen and hydrophobic interactions and showed enhanced binding with DHODH protein.

### Scaffold analysis

**Fig. 3:**
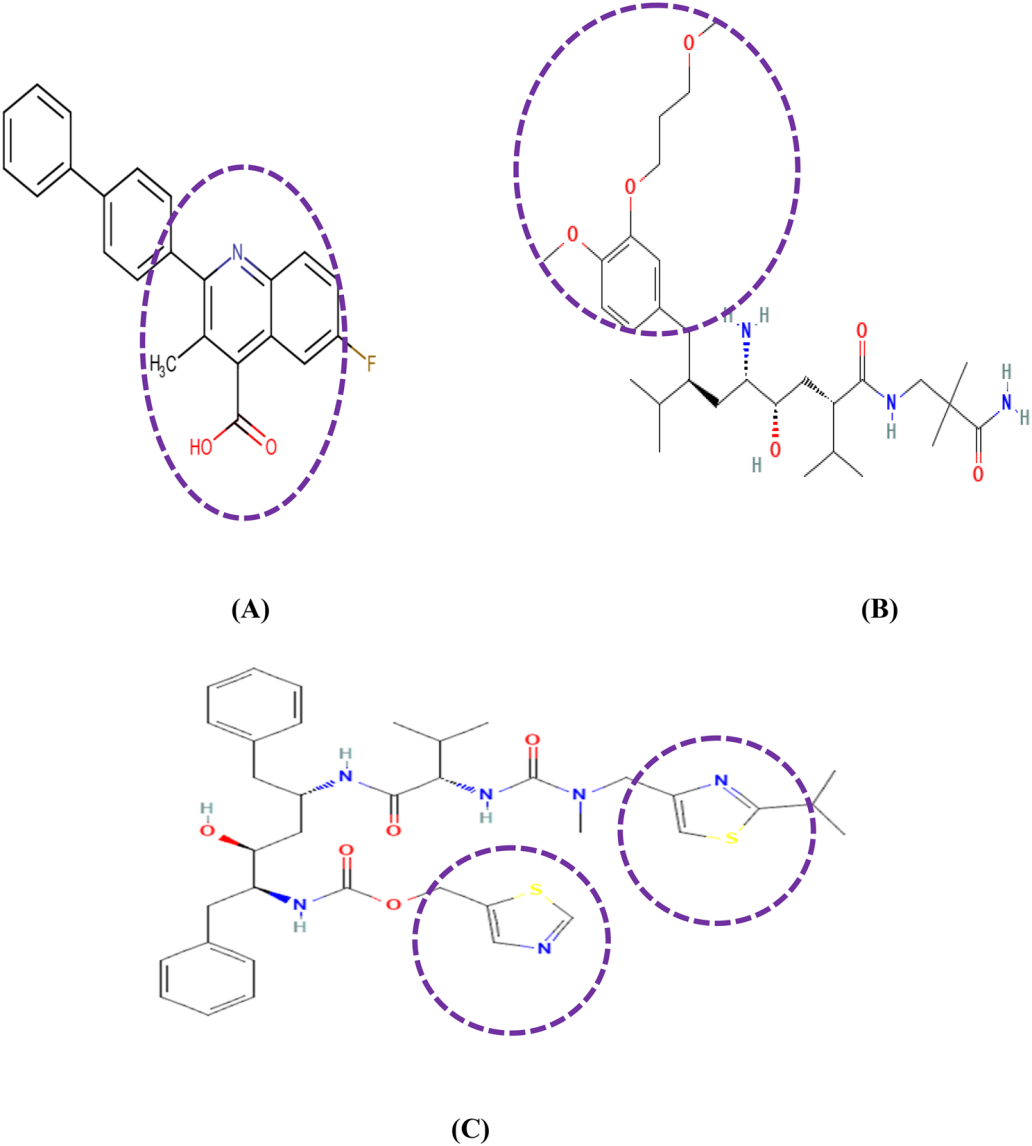
Scaffold analysis of **a**) Reference-brequinar with Quinoline carboxylic acid moiety; **b**) DB09026 Aliskiren with 4-methoxy-3-(3-methoxypropoxy) phenyl moiety and **c**) DB00503 Ritonavir with Thiazole moiety

Past studies indicate that brequinar has a quinoline-4-carboxylic acid moiety as the biological activity and is present in different natural and synthetic products. Many secondary metabolites contain quinoline, a heterocyclic molecule made up of pyridine and benzene rings [40]. The screened hit aliskiren (DB09026) contains a phenyl group with 3-methoxypropoxy group showing antibacterial and antifungal activities that enhance the microbial activity [41]. Similarly, Aliskiren is a mono methoxybenzene-based molecule, characterized by the presence of a 3-methoxypropoxy group. Mono methoxybenzene scaffolds are well-recognized for their broad spectrum of bioactive properties, including antiviral and anticancer activities. Recent studies had reported that a mono methoxybenzene derivative isolated from Zingiber officinale exhibited significant anticancer and anti-inflammatory effects. These findings further support the hypothesis that Aliskiren, through its structural features and scaffold similarity, holds promise as a potential anticancer agent, justifying its repurposing for oncology applications [42]. Furthermore, this drug is used as a direct inhibitor to reduce blood pressure by inhibiting renin and is also used for the treatment of cancer cachexia [43, 44]. Similarly, thiazole moieties of Ritonavir have been validated for diverse activities including antiviral, anticancer and anti-inflammatory properties [45–47]. The 2D structures of reference and the hit compounds emphasizing its scaffolds were visualized in Fig. 3.

### Molecular dynamic simulation

#### Conformational stability analysis

**Fig. 4:**
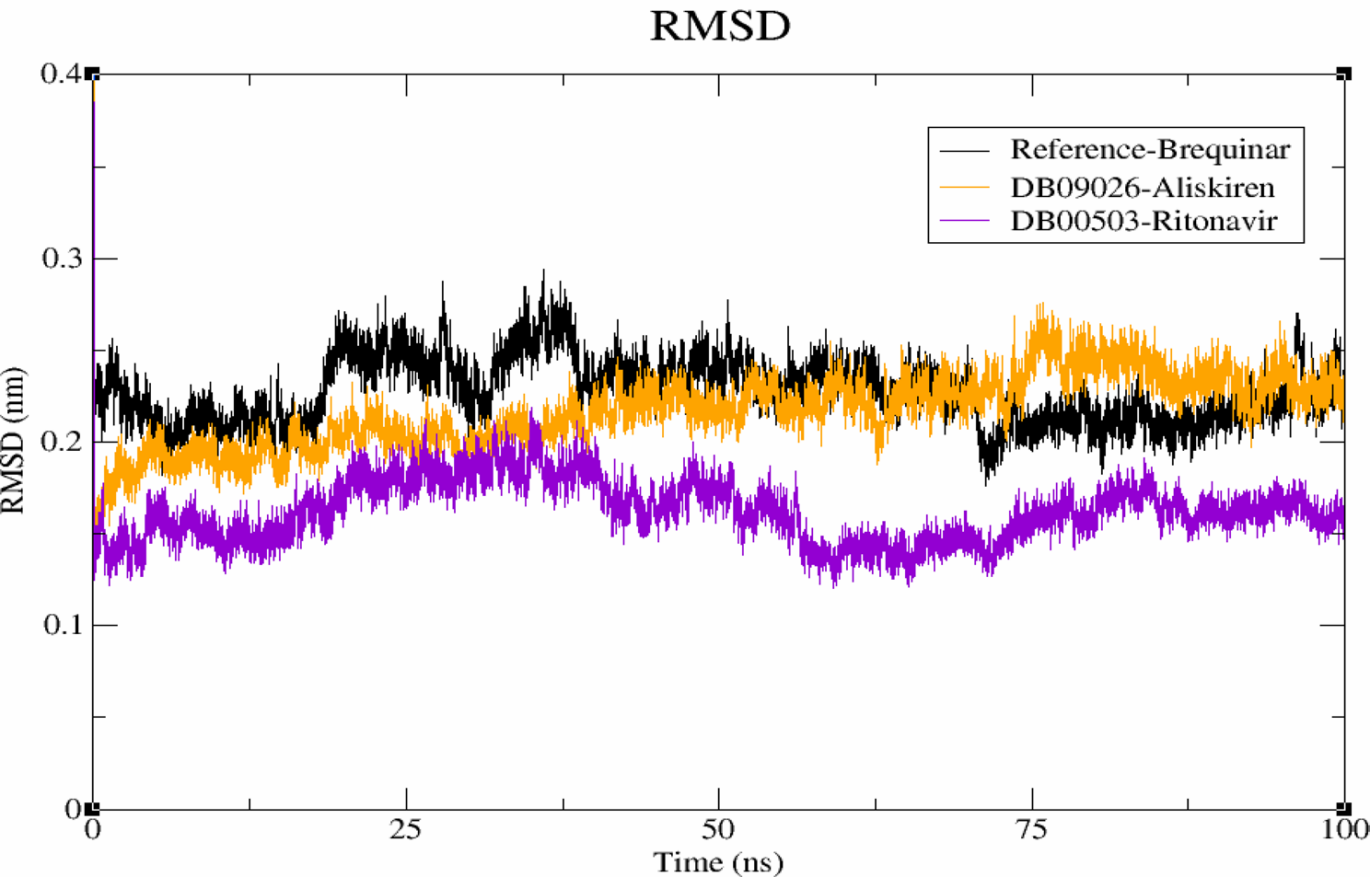
RMSD profiles of the protein–ligand complexes are shown, with brequinar as the reference (black), Aliskiren (DB09026) in orange, and Ritonavir (DB00503) in violet

Molecular Dynamic simulations are performed to identify the conformational properties of protein-ligand complex [48]. RMSD analysis is performed to examine the stability profiles of the two lead compounds in complex with DHODH, as well as the DHODH- brequinar complex. The RMSD plots of DHODH-brequinar (black), DHODH-DB09026 (orange), DHODH-DB00503 (violet) complexes are given in Fig. 4. Brequinar (black) showed a deviation at 20 ns and 40 ns, stabilizing at 0.25 nm until 75 ns. Similarly, DB09026 (Orange) exhibited a 0.2 nm deviation at the beginning, with a maximum deviation of 0.25 nm observed at 75 ns. DB00503 exhibited the minimum deviation at 60 ns. At the end of 100 ns simulation, the average RMSD values for brequinar, DB09026, and DB00503 were found to be 0.25 nm, and 0.15 nm, respectively. Thus, the binding of hits resulted in the least conformational change.

#### Residual flexibility analysis

**Fig. 5:**
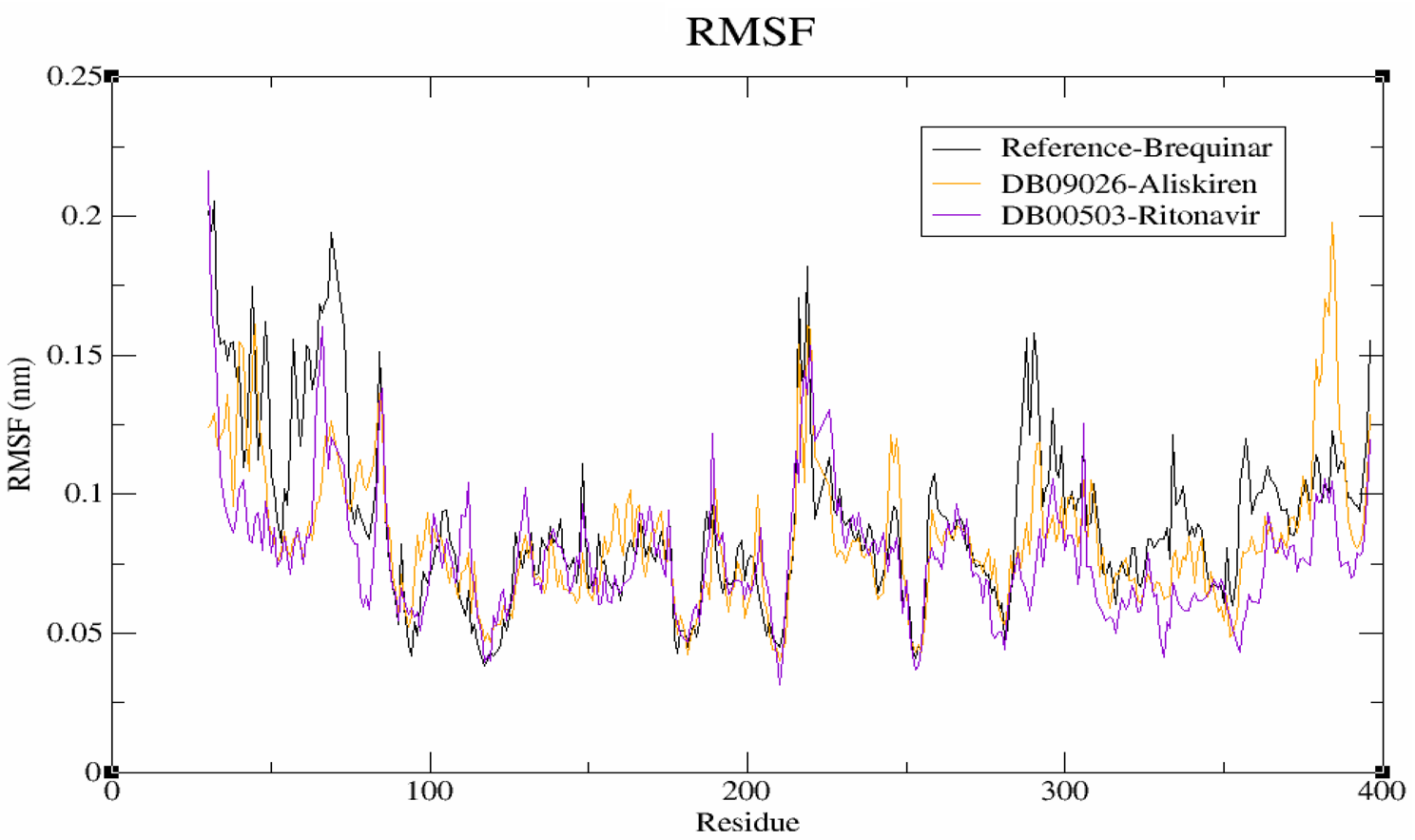
Root mean square fluctuation (RMSF) profiles of the protein–ligand complexes. Brequinar (reference) is shown in black, Aliskiren (DB09026) in orange, and Ritonavir (DB00503) in violet

The flexibility of protein-ligand complexes was evaluated using RMSF analysis. Figure 5 shows that the backbone fluctuation pattern of the protein-ligand complex. Notably, the binding residues (ARG136, GLN47, TYR356, THR360, LEU46, ALA55, HIS56, ALA59, PHE62, ASN145, LYS100, LEU68, LYS255 and PRO364) showed a lower average RMSF in DB09026 (0.12 nm) and DB00503 (0.11 nm) complexes than brequinar (0.15 nm) system at the end of a 100 ns. The research suggests that a higher RMSF number indicates less stability of the complex, whereas a lower RMSF value indicates greater stability [48]. Overall, the results indicate that the hits showed less fluctuation adapts the favourable position than brequinar at the binding site of the DHODH protein.

#### Hydrogen bond interaction

**Fig. 6:**
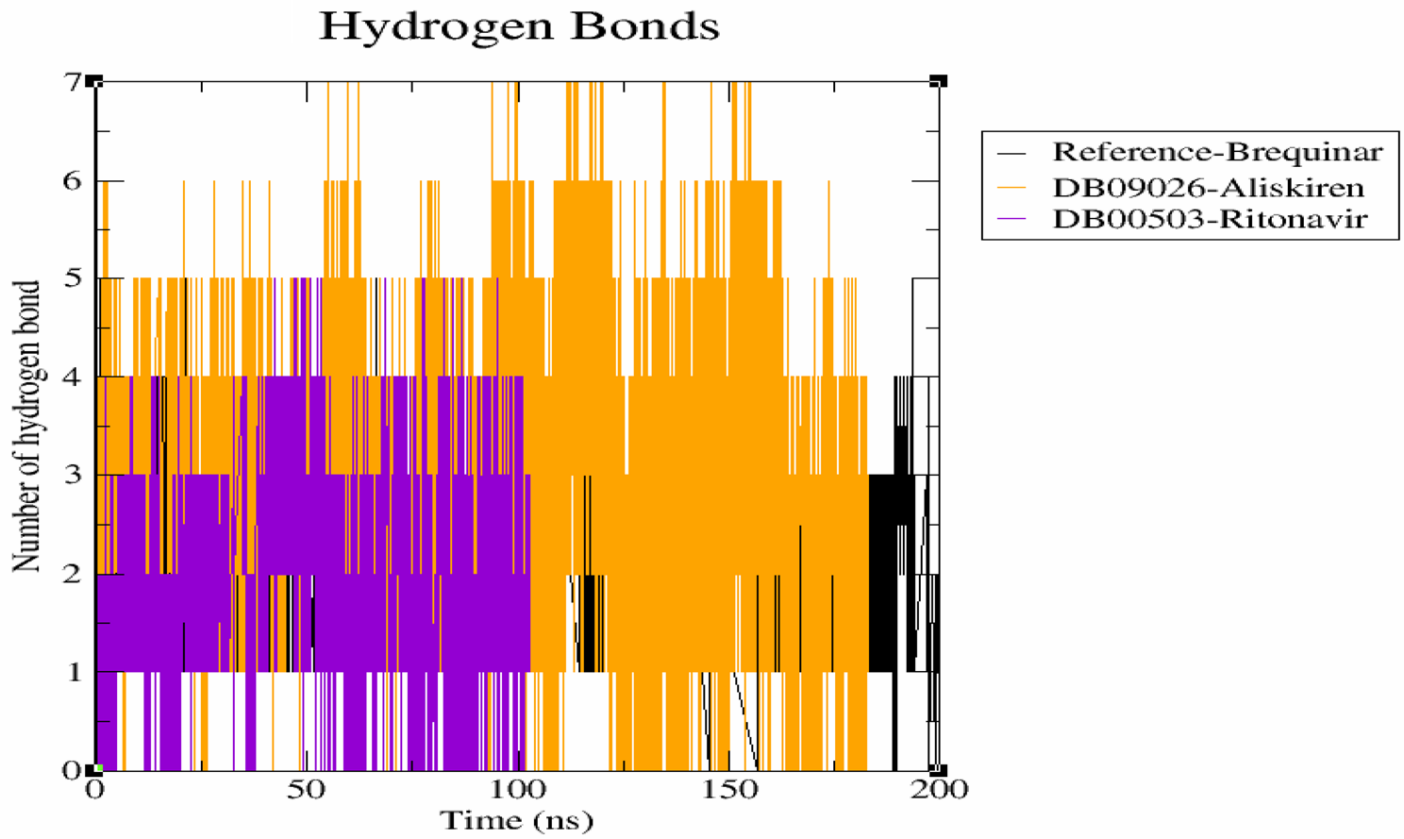
Hydrogen bond (H-bond) profiles of the protein–ligand complexes, with Brequinar (reference) represented in black, Aliskiren (DB09026) in orange, and Ritonavir (DB00503) in violet

Molecular recognition depends on the specificity and directionality of interactions made possible by the hydrogen bonding connection between the protein-ligand complexes. The gmx hbond tool of GROMACS was used to find the total number of hydrogen bonds between the DHODH ligand complexes [49]. The hydrogen bond plot (Fig. 6) revealed that the DHODH-brequinar, forms 3 hydrogen bonds. Meanwhile, DHODH-DB09026 and DHODH-DB00503 observed to exhibit exhibits 5–6 hydrogen bond interaction. Based on these results, we can conclude that both DB09026 and DB00503 complexes actively forms hydrogen bonds with active site residues of DHODH protein.

#### Compactness evaluation

**Fig. 7:**
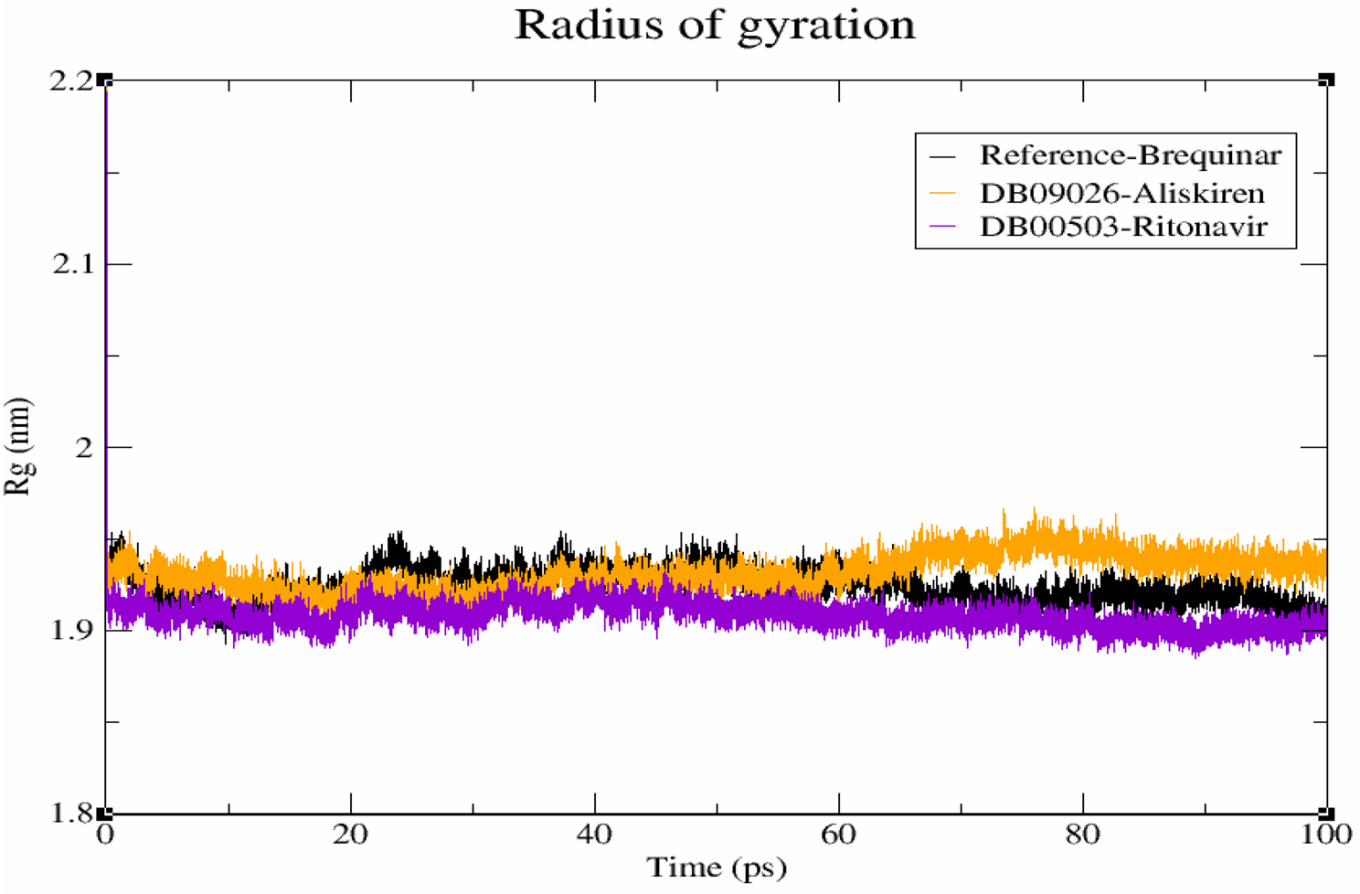
Radius of Gyration (Rg) profiles of the protein–ligand complexes. Brequinar (reference) is shown in black, Aliskiren (DB09026) in orange, and Ritonavir (DB00503) in violet

The compactness level of hits in the DHODH binding pocket were determined using degree of compactness (Rg). The weighted RMSD of each atom, as established by its centre of mass, is commonly denoted by Rg. Of note, an increase in Rg value indicates a decrease in protein structural compactness, leading to greater flexibility and instability [50]. Figure 7 shows the compactness of DHODH-brequinar, DHODH-DB09026 and DHODH-DB00503, which were found to be 1.93 nm, 1.94 nm and 1.92 nm, respectively, at the end of the 100 ns simulation. Finally, compactness evaluation reveals that hits adapts favourable conformation in the DHODH protein than reference molecule in our analysis.

#### Solvent accessible surface area

**Fig. 8:**
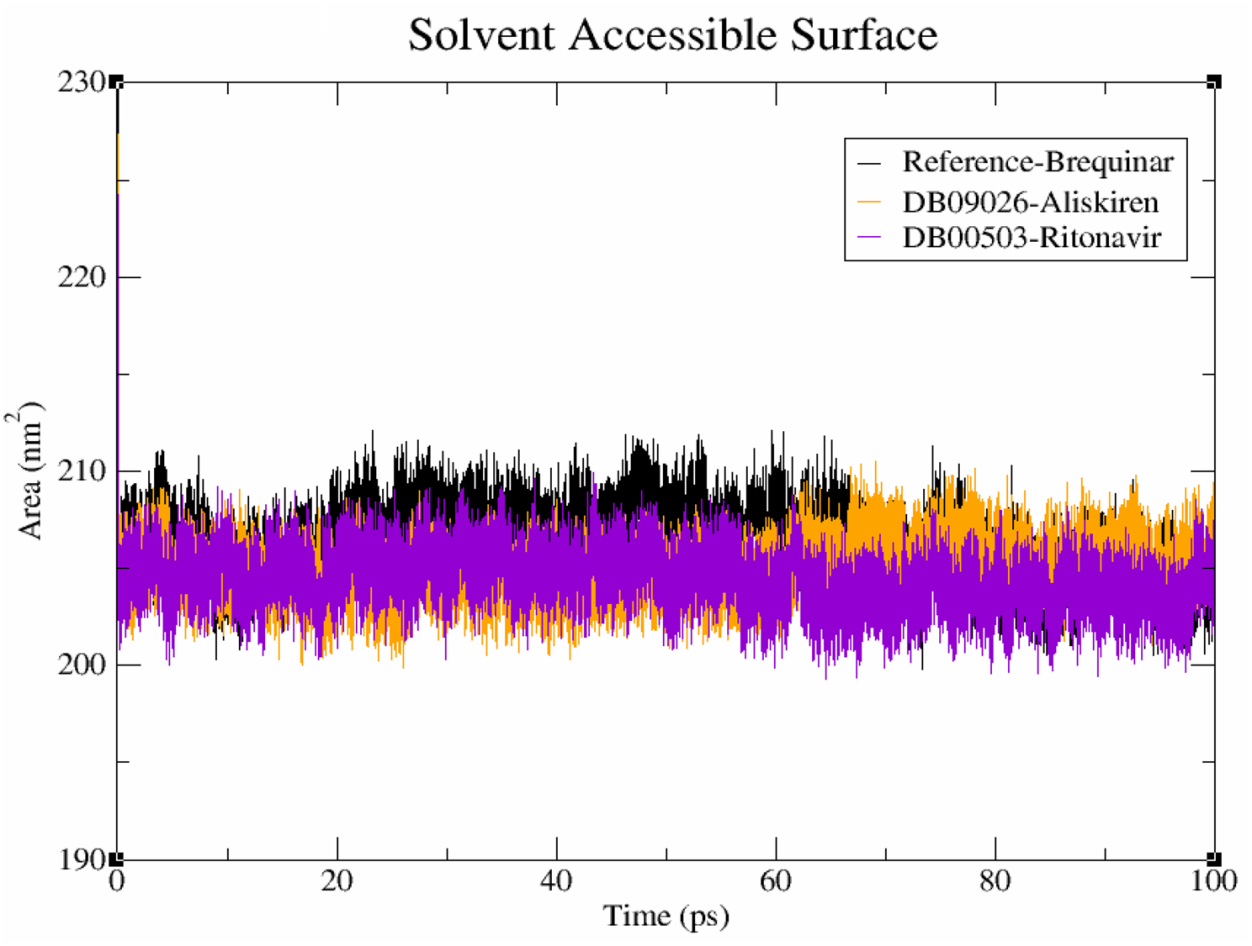
Solvent accessible surface area (SASA) profiles of the protein–ligand complexes. Brequinar (reference) is depicted in black, Aliskiren (DB09026) in orange, and Ritonavir (DB00503) in violet

The solvent-accessible surface area (SASA) method is used to calculate the surface area of polar and non-polar molecules in order to comprehend how residues interact with the surrounding solvent [15]. The SASA for the brequinar, DB09026, and DB00503 complexes is displayed to be 208 nm^2^, 209 nm^2^, and 208 nm^2^ respectively (Fig. 8). It is observed that both DB09026 and DB00503 complex with protein have stable conformational changes as of brequinar. There is no significant variation observed in terms of solvent accessible surface area analysis.

## Discussion

The DHODH is the plays a vital role in the *de novo* pyrimidine biosynthesis pathway and mitochondrial function. The overexpression of DHODH contributes to carcinogenesis and thus is considered as potential target for the cancer therapeutics. Here, libraries of 2619 FDA-approved compounds were exploited for its activity against DHODH through high-throughput strategy. Notably, two tiers of docking and binding free energy analysis were implemented to improve the prediction accuracy. The results yielded 51 compounds with satisfactory docking and binding energy values. It is also note that van der Waals interaction provides adequate involvement in the binding of inhibitors. MM/GB(PB)SA, is one of the most popular method, achieved reasonable correlation with experimental affinities was employed to validate the accuracy of the docking results [21]. Notably, Uni-GBSA, the MM/GBSA implementation used in our study, has demonstrated a Pearson correlation coefficient of 0.64 and RMSE of 1.70 kcal/mol in comparison to experimental affinities, supporting its robustness for predicting ΔG_bind_. Such approaches help narrow down viable candidates for downstream experimental validation and therapeutic exploration [51]. Following this rescoring, 17 lead compounds were selected based on their projected binding free energy. The toxicity and anti-tumor activity assessments for these 17 lead compounds were also found to be in the acceptable range.

The 17 lead compounds were categorized based on their functions concerning the DHODH and their hydrogen bond interactions with the active residues. Compounds such as DB00654 (Latanoprost), DB00944 (Demecarium), DB01100 (Pimozide), DB14185 (Aripiprazole Lauroxil), DB00137 (Lutein), DB06695 (Dabigatran Etexilate), DB09030 (Vorapaxar), DB00481 (Raloxifene) and DB06401 (Bazedoxifene) fail to form hydrogen bond interactions with important residues including ARG136, GLN47, THR360, and TYR356 in the protein’s binding pocket. Hence these nine compounds are not taken to the further scaffold analysis. However, DB00947 (Fulvestrant), DB06249 (Arzoxifene), DB08909 (Glycerol Phenylbutyrate), DB08964 (Gemeprost) and DB00549 (Zafirlukast) are able to maintain interaction only with TYR356. Hence, the lack of involvement of other key residues limits their binding strength. Further, DB13615 (Mifamurtide) higher molecular weight significantly impacts the consideration of the compound for further analysis due to poor permeability and low solubility. Adding together, the pharmacokinetic and dynamic properties are also not satisfactory.

On the other hand, key residues such as ARG136 and TYR356 interact with aliskiren, demonstrating its potential to inhibit DHODH. Precisely, TYR356 forms two hydrophobic interactions, indicating stronger binding with the protein and suggesting its potential as a promising target for DHODH in cancer treatment. Furthermore, the binding pattern of DHODH-DB00503 complex reveals extensive molecular interactions. Specifically, it forms seven hydrogen bonds with ASN145, TYR356, LYS255, GLY335 and THR357. In addition, ten hydrophobic interactions are established with ALA55, ALA59, VAL143, THR360, TYR356, TYR147, and PHE149. Among these, TYR356 emerges as key residue due to its higher number of hydrogen bonds, contributing significantly to the stability of the complex.

Aliskiren (DB09026) modulates blood pressure through the RAAS system (renin-angiotensin-aldosterone system), a hormonal system involved in regulating blood pressure and fluid balance system. Interestingly, RAAS, is known to promote cell proliferation, hypertrophy, and inflammation by activating signaling pathways linked through various mechanisms, particularly by influencing cell proliferation, mitochondrial function and nucleotide metabolism [52]. Since DHODH is dependent on mitochondrial electron transport, RAAS activation may modulate pyrimidine synthesis through mitochondrial dysfunction or oxidative stress.

A HIV protease inhibitor called Ritonavir (DB00503) inhibits viral proteinase enzyme that normally cleaves the structural and replicative proteins that are produced by the main HIV genes, including gag and pol [47]. Usually, viruses replication depends on pyrimidine nucleotides for their genome replication. Ritonavir also has off-target effects on cellular metabolism, particularly mitochondrial pathways which could interact with DHODH mechanism through inhibition [53].

In recent studies shows that the ritonavir act as a pharmacokinetic booster due to its potent and irreversible inhibitory effects on the CYP3A4 and also it inhibits drug transporters such as breast cancer resistance protein [54]. Aliskiren also shows anti-atherosclerotic and cardioprotective effects. It also has ability to reduce the reactive oxygen species (ROS) production. Aliskiren reduced tumour necrosis factor-α and interleukin-6 levels and inhibited myeloperoxidase activity, providing anti-inflammatory actions. Aliskiren also demonstrated antiproliferative efficacy by lowering epidermal hyperplasia and proliferating nuclear antigen levels. Similarly, ritonavir combined with docetaxel have increased antitumour effects in prostate and in breast cancers. It also acts as the antiproliferative agent in the ovarian, lung, bladder and pancreatic cancers [55–58]. Ours is the first report on ritonavir inhibitory effects in colorectal cancer. All these information highlights the hit compounds potential to inhibit DHODH in cancer cells. We, further hypothesize that combining the hit molecules with DHODH inhibitors could amplify anticancer effects by restricting pyrimidine biosynthesis.

The potential side effects of ritonavir have seen in the epithelium kidney cells induces endoplasmic reticulum stress and mitochondria stress. Similarly, aliskiren is known to cause kidney injury due to its RAAS blockade by angiotensin receptor blockers [58]. Although reports suggest that both compounds may exhibit minimal side effect, it could be of interesting choice for the management of cancer due to its improved potency. Indeed, the key limitation is lies in the lack of experimental validation of these compounds, which remains essential and could serve as an interesting future direction towards the development of novel DHODH inhibitor.

## Conclusion

The present study aimed to identify novel and potent DHODH inhibitors through virtual screening, with a distinct focus on repurposing. Initially, the binding poses of the target protein upon the ligand binding were analyzed using molecular docking and binding free energy calculations. Furthermore, the 17 lead compounds showed a higher binding free energy and was investigated for its toxicity, anti-cancer activity and scaffold analysis. Collective evidences from our analysis highlight satisfactory profile of DB09026 and DB00503 in all the investigation. The scaffold analysis reiterated the fact of anti-cancer activity of the identified compounds. Towards the end, the molecular dynamic simulation revealed comfortable positioning of the compounds in the binding pocket DHODH protein. However, experimental evidences on these compounds are certainly needed and likely to be an interesting future direction to confirm their activity against cancer cell lines.

## Data Availability

No datasets were generated or analysed during the current study.

## Abbreviations

DHODH: Dihydroorotate dehydrogenase
FDA: Food and Drug Administration
LEF: Leflunomide
TFM: Teriflunomide
RA: Rheumatoid Arthritis
MS: Multiple Sclerosis
MM: GBSA–Molecular Mechanics Generalised Born Surface Area
RMSD: Root Mean Square Deviation
RMSF: Root Mean Square Fluctuation
Rg: Radius of gyration
SASA: Solvent Accessible Surface Area
RAAS: Renin–Angiotensin–Aldosterone System
HIV: Human Immunodeficiency Virus

## References

[1] Yang X, Li C, Gou K, Liu X, Zhou Y, Zou J, Chen Q, Luo Y, Zhao Y. A novel and potent dihydroorotate dehydrogenase inhibitor suppresses the proliferation of colorectal cancer by inducing mitochondrial dysfunction and DNA damage. Med Comm–Oncology. 2022;1(1):e6. 10.1002/mog2.6.

[2] Kawatani M, Aono H, Hiranuma S, Shimizu T, Muroi M, Nogawa T, Ohishi T, Ohba SI, Kawada M, Yamazaki K, Dan S. Identification of a dihydroorotate dehydrogenase inhibitor that inhibits cancer cell growth by proteomic profiling. Oncol Res. 2023;31(6):833. 10.32604/or.2023.030241. 37744270 10.32604/or.2023.030241 PMC10513951

[3] Wang W, Cui J, Ma H, Lu W, Huang J. Targeting pyrimidine metabolism in the era of precision cancer medicine. Front Oncol. 2021;11:684961. 10.3389/fonc.2021.684961. 34123854 10.3389/fonc.2021.684961 PMC8194085

[4] Khairy A, Hammoda HM, Celik I, Zaatout HH, Ibrahim RS. Discovery of potential natural dihydroorotate dehydrogenase inhibitors and their synergism with brequinar via integrated molecular docking, dynamic simulations and in vitro approach. Sci Rep. 2022;12(1):19037. 10.1038/s41598-022-23006-1. 36351991 10.1038/s41598-022-23006-1 PMC9646789

[5] Galati S, Sainas S, Giorgis M, Boschi D, Lolli ML, Ortore G, Poli G, Tuccinardi T. Identification of human dihydroorotate dehydrogenase inhibitor by a pharmacophore-based virtual screening study. Molecules. 2022;27(12):3660. 10.3390/molecules27123660. 35744791 10.3390/molecules27123660 PMC9228440

[6] Abdel-Magid AF. Use of dihydroorotate dehydrogenase inhibitors for treatment of autoimmune diseases and cancer. ACS Med Chem Lett. 2020;11(11):2072–4. 10.1021/acsmedchemlett.0c00466. 33214811 10.1021/acsmedchemlett.0c00466 PMC7667645

[7] Wang S, Li Y, Lin Y, Li J, Guo L, Wang H, Lin X, Liu Z, Zhang B, Liao Z, Zhang Z. Bioinformatics analysis and experimental verification of the cancer-promoting effect of DHODH in clear cell renal cell carcinoma. Sci Rep. 2024;14(1):11985. 10.1038/s41598-024-62738-0. 38796629 10.1038/s41598-024-62738-0 PMC11127953

[8] Meng J, Zhang L, He Z, Hu M, Liu J, Bao W, Tian Q, Feng H, Liu H. Development of a machine learning-based target‐specific scoring function for structure‐based binding affinity prediction for human dihydroorotate dehydrogenase inhibitors. J Comput Chem. 2025;46(1):e27510. 10.1002/jcc.27510. 39325045 10.1002/jcc.27510

[9] Zhang L, Zhang J, Wang J, Ren C, Tang P, Ouyang L, Wang Y. Recent advances of human dihydroorotate dehydrogenase inhibitors for cancer therapy: current development and future perspectives. Eur J Med Chem. 2022;232:114176. 10.1016/j.ejmech.2022.114176. 35151222 10.1016/j.ejmech.2022.114176

[10] Cuthbertson CR, Guo H, Kyani A, Madak JT, Arabzada Z, Neamati N. The dihydroorotate dehydrogenase inhibitor brequinar is synergistic with ENT1/2 inhibitors. ACS Pharmacol Translational Sci. 2020;3(6):1242–52. 10.1021/acsptsci.0c00124. 10.1021/acsptsci.0c00124 PMC7737209 33344900

[11] Gehlot P, Kumar S, Kumar V, Vyas VK. Discovery of thiadiazoles as human dihydroorotate dehydrogenase (hDHODH) inhibitors by combined Structure-Based modelling methods. ChemistrySelect. 2024;9(10):e202304077. 10.1002/slct.202304077.

[12] Zuo Z, Liu X, Qian X, Zeng T, Sang N, Liu H, Zhou Y, Tao L, Zhou X, Su N, Yu Y. Bifunctional naphtho [2, 3-d][1, 2, 3] triazole-4, 9-dione compounds exhibit antitumor effects in vitro and in vivo by inhibiting dihydroorotate dehydrogenase and inducing reactive oxygen species production. J Med Chem. 2020;63(14):7633–52. 10.1021/acs.jmedchem.0c00512. 32496056 10.1021/acs.jmedchem.0c00512

[13] Kawatani M, Aono H, Shimizu T, Ohkura S, Hiranuma S, Muroi M, Ogawa N, Ohishi T, Ohba SI, Kawada M, Yamazaki K. Identification of dihydroorotate dehydrogenase inhibitors indoluidins that inhibit cancer cell growth. ACS Chem Biol. 2021;16(11):2570–80. 10.1021/acschembio.1c00625. 34730931 10.1021/acschembio.1c00625

[14] Li C, Zhou Y, Xu J, Zhou X, Huang Z, Zeng T, Yang X, Tao L, Gou K, Zhong X, Chen Q. A novel series of Teriflunomide derivatives as orally active inhibitors of human dihydroorotate dehydrogenase for the treatment of colorectal carcinoma. Eur J Med Chem. 2022;238:114489. 10.1016/j.ejmech.2022.114489. 35640328 10.1016/j.ejmech.2022.114489

[15] Dasmahapatra U, Kumar CK, Das S, Subramanian PT, Murali P, Isaac AE, Ramanathan K, Mm B, Chanda K. In-silico molecular modelling, MM/GBSA binding free energy and molecular dynamics simulation study of novel pyrido fused Imidazo [4, 5-c] Quinolines as potential anti-tumor agents. Front Chem. 2022;10:991369. 10.3389/fchem.2022.991369. 36247684 10.3389/fchem.2022.991369 PMC9566731

[16] Ramesh P, Veerappapillai S. Designing novel compounds for the treatment and management of RET-positive non-small cell lung cancer—fragment-based drug design strategy. Molecules. 2022;27(5):1590. 10.3390/molecules27051590. 35268691 10.3390/molecules27051590 PMC8911629

[17] Eberhardt J, Santos-Martins D, Tillack AF, Forli S. AutoDock Vina 1.2. 0: new Docking methods, expanded force field, and python bindings. J Chem Inf Model. 2021;61(8):3891–8. 10.1021/acs.jcim.1c00203. 34278794 10.1021/acs.jcim.1c00203 PMC10683950

[18] Goswami V, Patel D, Rohit S, Chaube U, Patel B. Homology modelling, binding site identification, molecular Docking and molecular dynamics simulation study of emerging and promising drug target of Wnt signaling–Human Porcupine enzyme. Results Chem. 2024;7:101482. 10.1016/j.rechem.2024.101482.

[19] Grotsch K, Sadybekov AV, Hiller S, Zaidi S, Eremin D, Le A, Liu Y, Smith EC, Illiopoulis-Tsoutsouvas C, Thomas J, Aggarwal S. Virtual screening of a chemically diverse superscaffold library enables ligand discovery for a key GPCR target. ACS Chem Biol. 2024;19(4):866–74. 10.1021/acschembio.3c00602. 38598723 10.1021/acschembio.3c00602 PMC12034570

[20] Murali P, Karuppasamy R. Exploration of natural product database for the identification of potent inhibitor against IDH2 mutational variants for glioma therapy. J Mol Model. 2023;29(1):6. 10.1007/s00894-022-05409-z. 10.1007/s00894-022-05409-z 36484830

[21] Aldeghi M, Heifetz A, Bodkin MJ, Knapp S, Biggin PC. Predictions of ligand selectivity from absolute binding free energy calculations. J Am Chem Soc. 2017;139(2):946–57. 10.1021/jacs.6b11467. 28009512 10.1021/jacs.6b11467 PMC5253712

[22] Yang M, Bo Z, Xu T, Xu B, Wang D, Zheng H. Uni-GBSA: an open-source and web-based automatic workflow to perform MM/GB (PB) SA calculations for virtual screening. Brief Bioinform. 2023;24(4):bbad218. 10.1093/bib/bbad218. 37328705 10.1093/bib/bbad218

[23] Khan MF, Ali A, Rehman HM, Noor Khan S, Hammad HM, Waseem M, Wu Y, Clark TG, Jabbar A. Exploring optimal drug targets through subtractive proteomics analysis and pangenomic insights for tailored drug design in tuberculosis. Sci Rep. 2024;14(1):10904. 10.1038/s41598-024-61752-6. 38740859 10.1038/s41598-024-61752-6 PMC11091173

[24] Desai A, Mahajan V, Ramabhadran RO, Mukherjee R. Binding order of substrate and cofactor in sulfonamide monooxygenase during Sulfa drug degradation: in silico studies. J Biomol Struct Dynamics. 2024 Jan 18:1–5. 10.1080/07391102.2024.2306495 10.1080/07391102.2024.2306495 38263732

[25] Vangone A, Schaarschmidt J, Koukos P, Geng C, Citro N, Trellet ME, Xue LC, Bonvin AM. Large-scale prediction of binding affinity in protein–small ligand complexes: the PRODIGY-LIG web server. Bioinformatics. 2019;35(9):1585–7. 10.1093/bioinformatics/bty816. 31051038 10.1093/bioinformatics/bty816

[26] Bandi J, Malkhed V, Nambigari N. An insilico study of KLK-14 protein and its Inhibition with Curcumin and its derivatives. Chem Pap. 2022;76(8):4955–66. 10.1007/s11696-022-02209-w.

[27] Murali P, Karuppasamy R. Exploring the potential of nutraceutical to combat gliomas: focus on mIDH2 protein. Front Phys. 2024;12:1345834. 10.3389/fphy.2024.1345834.

[28] Banerjee P, Eckert AO, Schrey AK, Preissner R. ProTox-II: a webserver for the prediction of toxicity of chemicals. Nucleic Acids Res. 2018;46(W1):W257–63. 10.1093/nar/gky318. 29718510 10.1093/nar/gky318 PMC6031011

[29] Al-Jarf R, de Sá AG, Pires DE, Ascher DB. pdCSM-cancer: using graph-based signatures to identify small molecules with anticancer properties. J Chem Inf Model. 2021;61(7):3314–22. 10.1021/acs.jcim.1c00168. 34213323 10.1021/acs.jcim.1c00168 PMC8317153

[30] Vishwakarma S, Hernandez-Hernandez S, Ballester PJ. Graph neural networks are promising for phenotypic virtual screening on cancer cell lines. Biology Methods Protocols. 2024;9(1):bpae065. 10.1093/biomethods/bpae065. 39502795 10.1093/biomethods/bpae065 PMC11537795

[31] Thirunavukkarasu MK, Veerappapillai S, Karuppasamy R. Computational biophysics approach towards the discovery of multi-kinase blockers for the management of MAPK pathway dysregulation. Mol Diversity. 2023;27(5):2093–110. 10.1007/s11030-022-10545-y. 10.1007/s11030-022-10545-y 36260173

[32] Suresh R, Karuppasamy R. Seaweed-based PPO inhibitors as a new frontier in biological weed control for sorghum cultivation: from ocean to field. Protoplasma. 2025 Mar 4:1–7. 10.1007/s00709-025-02049-x 10.1007/s00709-025-02049-x 40035808

[33] Liu S, Neidhardt EA, Grossman TH, Ocain T, Clardy J. Structures of human dihydroorotate dehydrogenase in complex with antiproliferative agents. Structure. 2000;8(1):25–33. 10.1016/S0969-2126(. 00)00077 – 0. 10673429 10.1016/s0969-2126(00)00077-0

[34] Liu X, Ren X, Ren X, Zhang J, Hua M, Sui C, Liu Z, Luo F, Ran S, Li X, Cui L. Discovery of a new class of Thiazolidin-4-one-Based inhibitors of human dihydroorotate dehydrogenase: biological activity Evaluation, molecular Docking, and molecular dynamics. ACS Omega. 2025;10(12):12393–402. 10.1021/acsomega.4c11459. 40191330 10.1021/acsomega.4c11459 PMC11966315

[35] Paranthaman P, Veerappapillai S. Identification of putative indoleamine 2, 3-dioxygenase 1 (IDO1) and tryptophan 2, 3-dioxygenase (TDO) dual inhibitors for triple-negative breast cancer therapy. J Biomol Struct Dynamics. 2025 Jan 18:1–9. 10.1080/07391102.2024.2332509 10.1080/07391102.2024.2332509 39861977

[36] Bray F, Laversanne M, Sung H, Ferlay J, Siegel RL, Soerjomataram I, Jemal A. Global cancer statistics 2022: GLOBOCAN estimates of incidence and mortality worldwide for 36 cancers in 185 countries. Cancer J Clin. 2024;74(3):229–63. 10.3322/caac.21834. 10.3322/caac.21834 38572751

[37] Antony A, Karuppasamy R. Searching of novel herbicides for paddy field weed management—a case study with acetyl-CoA carboxylase. Agronomy. 2022;12(7):1635. 10.3390/agronomy12071635.

[38] Coimbra JT, Feghali R, Ribeiro RP, Ramos MJ, Fernandes PA. The importance of intramolecular hydrogen bonds on the translocation of the small drug Piracetam through a lipid bilayer. RSC Adv. 2021;11(2):899–908. 10.1039/d0ra09995c. 35423709 10.1039/d0ra09995c PMC8693363

[39] Savla R, Browne J, Plassat V, Wasan KM, Wasan EK. Review and analysis of FDA approved drugs using lipid-based formulations. Drug Dev Industrial Pharm. 2017 Nov 2;43(11):1743–58 10.1080/03639045.2017.1342654 10.1080/03639045.2017.1342654 28673096

[40] Fikriya SH, Cahyana AH. Study of antioxidant activity of the derivatives of quinoline-4-carboxylic acids by the modification of Isatin via Pfitzinger reaction. Makara J Sci. 2023;27(2):9. 10.7454/mss.v27i2.1394.

[41] Zarrabi N. In vitro evaluation of antibacterial and antifungal properties of some new 1, 3, 4-oxadiazole derivatives containing phenyl group. Infect Epidemiol Microbiol. 2020;6(3):177–92. 10.29252/iem.6.3.177.

[42] Kim TW, Lee HG. Anti-Inflammatory 8-Shogaol mediates apoptosis by inducing oxidative stress and sensitizes radioresistance in gastric cancer. Int J Mol Sci. 2024;26(1):173. 10.3390/ijms26010173. 39796030 10.3390/ijms26010173 PMC11719885

[43] Buczko W, Hermanowicz JM. -Pharmacokinetics and pharmacodynamics of aliskiren, an oral direct Renin inhibitor. Pharmacol Rep. 2008;60(5):623. PMID: 19066408. 19066408

[44] Wang C, Guo D, Wang Q, You S, Qiao Z, Liu Y, Dai H, Tang H. Aliskiren targets multiple systems to alleviate cancer cachexia. Oncol Rep. 2016;36(5):3014–22. 10.3892/or.2016.5118. 27667116 10.3892/or.2016.5118

[45] Kushwaha P, Pandey S. 1, 3-thiazole derivatives as a promising scaffold in medicinal chemistry: a recent overview. Anti-Inflammatory & Anti-Allergy Agents in Medicinal Chemistry Rent Medicinal Chemistry-Anti-Inflammatory and Anti-Allergy Agents. 2023 Sep 1;22(3):133–63. 10.2174/0118715230276678231102150158 10.2174/0118715230276678231102150158 37997807

[46] Petrou A, Fesatidou M, Geronikaki A. Thiazole ring—A biologically active scaffold. Molecules. 2021;26(11):3166. 10.3390/molecules26113166. 34070661 10.3390/molecules26113166 PMC8198555

[47] Maggisano V, Gargano A, Maiuolo J, Ortuso F, De Amicis F, Alcaro S, Bulotta S. Rational identification of Ritonavir as IL-20 receptor A ligand endowed with antiproliferative properties in breast cancer cells. Int J Mol Sci. 2025;26(3):1285. 10.3390/ijms26031285. 39941053 10.3390/ijms26031285 PMC11818535

[48] Krishnamoorthy HR, Karuppasamy R. A multitier virtual screening of antagonists targeting PD-1/PD-L1 interface for the management of triple-negative breast cancer. Med Oncol. 2023;40(11):312. 10.1007/s12032-023-02183-7. 37777635 10.1007/s12032-023-02183-7

[49] Murali P, Karuppasamy R. Imidazole and biphenyl derivatives as anti-cancer agents for glioma therapeutics: computational drug repurposing strategy. Anti-Cancer Agents Med Chemistry-Anti-Cancer Agents. 2023;23(9):1085–101. 10.2174/1871520623666230125090815. 10.2174/1871520623666230125090815 36698225

[50] Ramesh P, Karuppasamy R, Veerappapillai S. Machine learning driven drug repurposing strategy for identification of potential RET inhibitors against non-small cell lung cancer. Med Oncol. 2022;40(1):56. 10.1007/s12032-022-01924-4. 36542155 10.1007/s12032-022-01924-4 PMC9769489

[51] Wang F, Zhou W, Yang M, Niu J, Huang W, Chen Z, Chen Y, Wang D, Zhang J, Wu S, Yan S. Structure-guided discovery of novel AflG inhibitors for aflatoxin contamination control in Aspergillus flavus. Front Microbiol. 2024;15:1425790. 10.3389/fmicb.2024.1425790. 39070265 10.3389/fmicb.2024.1425790 PMC11272468

[52] Hassani B, Attar Z, Firouzabadi N. The renin-angiotensin-aldosterone system (RAAS) signaling pathways and cancer: foes versus allies. Cancer Cell Int. 2023;23(1):254. 10.1186/s12935-023-03080-9. 37891636 10.1186/s12935-023-03080-9 PMC10604988

[53] Zheng Y, Li S, Song K, Ye J, Li W, Zhong Y, Feng Z, Liang S, Cai Z, Xu K. A broad antiviral strategy: inhibitors of human DHODH pave the way for host-targeting antivirals against emerging and re-emerging viruses. Viruses. 2022;14(5):928. 10.3390/v14050928. 35632670 10.3390/v14050928 PMC9146014

[54] Loos NH, Beijnen JH, Schinkel AH. The inhibitory and inducing effects of ritonavir on hepatic and intestinal CYP3A and other drug-handling proteins. Biomed Pharmacother. 2023 Jun 1;162: 10.1016/j.biopha.2023.114636 10.1016/j.biopha.2023.114636 PMC10065864 37004323

[55] Barta A, Cebova M, Kovac A, Koneracka M, Zavisova V, Pechanova O. Aliskiren-loaded nanoparticles downregulate (Pro) Renin receptor and ACE gene expression in the heart of spontaneously hypertensive rats: effect on NADPH oxidase. Int J Mol Sci. 2024;25(2):846. 10.3390/ijms25020846. 38255922 10.3390/ijms25020846 PMC10815459

[56] Pereira M, Vale N. Ritonavir’s evolving role: A journey from antiretroviral therapy to broader medical applications. Curr Oncol. 2024;31(10):6032–49. 10.3390/curroncol31100450. 39451754 10.3390/curroncol31100450 PMC11505664

[57] Pawloski PL, Moreira CG, Horinouchi CD, Fernandes D, Júnior LR, Machado W, Cabrini DA, Dietrich M, Paludo K, Otuki MF. Aliskiren: preclinical evidence for the treatment of hyperproliferative skin disorders. Biomed Pharmacother. 2018;104:151–7. 10.1016/j.biopha.2018.03.157. 29772435 10.1016/j.biopha.2018.03.157

[58] Zheng SL, Roddick AJ, Ayis S. Effects of Aliskiren on mortality, cardiovascular outcomes and adverse events in patients with diabetes and cardiovascular disease or risk: A systematic review and meta-analysis of 13,395 patients. Diabetes Vascular Disease Res. 2017;14(5):400–6. 10.1177/1479164117715854. 10.1177/1479164117715854 PMC5600262 28844155

